# Water Drinking in the Treatment of Chronic Constipation

**Published:** 1909-05-22

**Authors:** 


### Medicine.

### WATER DRINKING IN THE TREATMENT OF CHRONIC CONSTIPATION.

There can be little doubt that the two most important points in the prevention or cure of chronic constipation are, first, the persistent encouragement and training of the diurnal habit of emptying the bowel at a regular hour; and, secondly, the avoidance of interference with this diurnal habit by the thoughtless prescription of laxatives or purgatives, which temporarily relieve a constipation but which tend to encourage its recurrence.

A third very important point, however, is the use of water-drinking—infinitely preferable to purgative or cathartic drugs. This is no new form of treatment—it is as old as is Spa treatment of any kind; nevertheless, there are features and details in it that receive too little attention as a rule.

Water is healthful, but persons often drink too much, or take it at too high or too low a temperature. In the treatment of constipation indiscriminate water-drinking should be discountenanced. Consequently, when this therapeutic agent is prescribed it should be ordered with details as to time, quantity, temperature, relation to food, and so on, with a definite object in view according to the case. Except in special instances, water should never be ingested at temperatures below 50° F. or above 130° F., positive harm being sometimes done to the gastro-intestinal mechanism when either of these extremes is passed. It is rarely necessary to drink more than from ten to fifteen glasses of water during the day, and usually from five to six are ample.

The effect upon the stomach and intestine is much better when a small quantity, such as half a tumblerful, is taken at frequent intervals—say every hour or every two hours—than when the same amount of water is consumed at two or three draughts. It has been definitely proved that the stomach absorbs hardly any water, the small intestine little, and the colon a great deal. Owing to the non-absorption of water by the stomach, if a large quantity is swallowed it interferes with digestion, distends the stomach, and by its weight causes sagging of this viscus, favouring gastroptosis and enteroptosis, which in their turn aggravate the constipated state. Ordinarily, constipated subjects should drink from one glass to a glass and a half of water on rising, and lesser amounts at short intervals during the day.

The habit of drinking several glasses of water, particularly of iced water, at meal-time is detrimental to health, because the water washes the food down before it has been properly masticated, dilutes the gastric contents unduly, upsets the local circulation, and generally interferes with the digestive process. Indigestion, gastrectasis, constipation, anæmia, and other ailments are liable to ensue. On the other hand, drinking cool or cold water in moderation at meal-times has a healthy influence on the alimentary canal. Drinking of cold water is contra-indicated in colic, in the treatment of very old and feeble persons, in cases of gastric ulcer, arteriosclerosis, persistent anæmia, diseases of the kidney, and in persons who are from any cause either physically fatigued or hot and perspiring.

Cool water produces a general tonic effect and stimulates the intestinal musculature and mucous glands to greater activity. Warm water has an opposite effect. It soothes and quiets the alimentary tract, relieves irritation and pain, and arrests or diminishes enterospasm.

The beneficent effect of water in constipated subjects is due as much, if not more, to its thermic as to its mechanical and solvent action. Many persons suffering from severe obstipation have been cured without drugs by judicious water-drinking, together with attention to diet and with efforts on the patient's part to secure a daily evacuation after the morning meal.

When chronic biliousness and signs of auto-intoxication are present, a glass and a half of cold water taken in the morning on an empty stomach stimulates the stomach, liver, and gastro-intestinal tract, and in this way helps to regulate the stools and eliminate toxins from the bowel.

Drinking of warm and moderately hot water should be discouraged in persons afflicted with chronic habitual atonic costiveness, for the reason that a lethargic state and infrequent evacuations are encouraged by its sedative action. Water at about body temperature—96° F. to 100° F.—acts similarly, but to a less marked degree. Very hot water—125° F. to 140° F.—stimulates peristalsis, but it prejudices the gastric mucosa locally; it should not be employed for long except when constipation is complicated by hypopepsia, gastro-succorrhœa, gastralgia, or enterospasm.

From what has been said it may be inferred that the temperature of the water to be taken by constipated patients should be decided with considerable care if the best effects of the treatment in the individual case are to be obtained. It may be stated that drinking of cold water is advisable in chronic atonic constipation, for the reason that it stimulates the local and general circulation, improves assimilaton and metabolism, favours elimination of toxins of all kinds through its effect upon the skin, liver, and kidneys, increases peristaltic action, causes a more abundant secretion of mucus for lubricating the bowel, and softens the fæces, so that the stools become normal in quantity and in frequency.

Cold drinks produce so much increase in intestinal contraction that pain sometimes results, and occasionally actual colic; on account of this tendency drinking of cold water should be prohibited in persons suffering from enterospasm, colic, or obstructive symptoms.

In spastic constipation the fæcal current is partially or completely blocked because of an occlusion due to simultaneous contraction of both the longitudinal and circular muscular fibres. Such enterospasms may occur at short or long intervals, and last for several minutes, hours, or days. Hot water —at 110° F. to 120° F.—taken by the mouth has almost a specific action in relieving and preventing constipation of this type. The hot-water drinking may be reinforced by hot abdominal fomentations, which act in a similar manner. In addition to its sedative action, hot water taken internally is as serviceable as cold water in liquefying the fæces, softening any scybala that may be present, and thus helping to overcome habitual constipation. Several cases have been recorded in which mechanical obstruction due to inspissation and impaction of fæces has been overcome by copious drinking of hot water. The good results obtained in this class of sufferers have been attributed to both the soothing effect of the hot water reducing intestinal irritability and muscular spasms, and its mechanical action in dislodging the cause of the obstruction.

